# Population genetics of 30 INDELs in populations of Poland and Taiwan

**DOI:** 10.1007/s11033-013-2521-7

**Published:** 2013-05-21

**Authors:** Witold Pepinski, Monica Abreu-Glowacka, Malgorzata Koralewska-Kordel, Eliza Michalak, Krzysztof Kordel, Anna Niemcunowicz-Janica, Michal Szeremeta, Magdalena Konarzewska

**Affiliations:** 1 Department of Forensic Medicine, Medical University of Bialystok, Bialystok, Poland; 2 Department of Forensic Medicine, Poznan University of Medical Sciences, Poznan, Poland; 3 Department of Medical Genetics, Warsaw Medical University, Warsaw, Poland

**Keywords:** INDEL polymorphism, Investigator DIPplex, Poland, Taiwan, Population genetics, Forensic efficiency

## Abstract

The Investigator DIPplex^®^ kit (Qiagen) contain components for the simultaneous amplification and analysis of 30 biallelic autosomal INDELs and amelogenin. The objective of this study was to estimate the diversity of the 30 markers in Polish (*N*_P_ = 122) and Taiwanese (*N*_T_ = 126) population samples and to evaluate their usefulness in forensic genetics. All amplicon lengths were shorter than 160 base pairs. The DIPplex genotype distributions showed no significant deviation from Hardy–Weinberg rule expectations (Bonferroni corrected) except for DLH39 in the Taiwanese population. Among the Poles and the Taiwanese the mean observed heterozygosity values are 0.4385 and 0.4079, and the combined matching probability values are 7.98 × 10^−14^ and 1.22 × 10^−11^, respectively. The investigated marker set has been confirmed as a potential extension to standard short tandem repeat-based kits or a separate informative system for individual identification and kinship analysis. Eight INDELs have been selected as possible ancestry informative single-nucleotide polymorphisms for further analyses.

## Introduction

INDELs (insertion–deletion) or DIPs (deletion–insertion polymorphisms) are short length diallelic polymorphisms, consisting of the presence or absence of short sequences (typically 1–50 bp). They are relatively common throughout the human genome representing 15–20 % of all polymorphisms [1] with the total number estimated at about 2 million [2]. Short amplicon size (50–150 bp), low mutation rate (<2 × 10^−8^), and capacity to multiplex (30–40 markers) and type using a single multiplexed PCR with fluorescently labeled primers followed by capillary electrophoresis (a current technology for human identification) [3–5] are the main advantages that make INDELs useful in forensic genetics applications including individual identification, kinship testing, population studies and ancient DNA analysis [6–8]. The Investigator DIPplex^®^ kit (Qiagen) contain components for the simultaneous amplification and analysis of 30 biallelic autosomal INDELs and amelogenin. The INDELs are distributed over 19 autosomes at the minimum distance of 10 Mbp to routinely used STR and SNP markers. The allele length variations of the INDELs are between 4 and 22 bp, and all amplicons are shorter than 160 bp.

## DNA extraction

Buccal swabs were anonymized and collected from unrelated volunteers along with information on the birthplace and ethnicity of the donor. Signed informed consents were obtained from all the participants and this study complied with the protocol approved by the Ethical Committee of Poznan University of Medical Sciences (Ref: 139/13). The population sample sizes were: Poles (*N*_P_ = 122), and Taiwanese (*N*_T_ = 126). The extraction of genomic DNA was carried out using QIAamp^®^ DNA Mini Kit (Qiagen). The quantitation was performed using Quantifiler™ Human DNA Quantification Kit on a 7500 Real-Time PCR System (Applied Biosystems) according to the manufacturer’s specifications. The samples were then normalized to 100 pg/μl and stored at −20 °C until amplification.

## Amplification and genotyping

PCR conditions were applied according to the protocol recommended by the manufacturer of the Investigator DIPplex Kit (Qiagen) in PCR System 9700 (Applied Biosystems, USA) with a total reaction volume adjusted to 5 μl containing 1.8 μl nuclease-free water, 1.0 μl reaction mix A, 1.0 μl primer mix, 0.2 μl MultiTaq2 polymerase, and 100 pg DNA template. Control DNA XY5 was used to test performance of the DIPplex Kit. The amplification was performed with 30 PCR cycles. Electrophoresis and typing were performed in 3130 Genetic Analyzer (Applied Biosystems, USA) using a 36 cm capillary array and a denaturing polymer POP-4. BTO 550 (Qiagen) was used as the internal lane standard spanning fragments from 60 to 550 bps. Prior to the analysis, a five dye matrix standard (BT5) was established with the fluorescent labels dyes 6-FAM, BTG, BTY, BTR, and BTO under the Any5Dye virtual filter. Samples were injected for 10 s at 3 kV and electrophoresed for 1000 s at 15 kV at a run temperature of 60 °C. The data were collected using Data Collection v3.0 software. GeneMapper^®^ ID-X v1.1.1 software was used for the INDELs classification.

## Statistical analysis

Estimates for genetic diversity (allele frequencies, heterozygosity), conformance to expectations of the Hardy–Weinberg equilibrium (HWE) and for independence (Linkage Disequilibrium, LD) were obtained using GDA v1.0 software [9]. For multiple comparisons, the original significance levels achieved (*P* values) were transformed by the Bonferroni correction procedure [10], i.e. 30 markers per database yield an actual significance level of 0.0016667. Forensic informativeness was estimated by calculating discrimination power (DP), match probability (MP), polymorphic information content (PIC), typical paternity index (TPI), and power of paternity exclusion (PE) using Powerstats v1.2 spreadsheet (Promega) [11]. Comparison of allele frequency distributions was performed by means of a pairwise population comparison test (R × C contingency test; G. Carmody, Ottawa, Canada). AMOVA and population differentiation exact test were calculated with the Arlequin v.3.5 software [12].

## Results and discussion

**Fig. 1:**
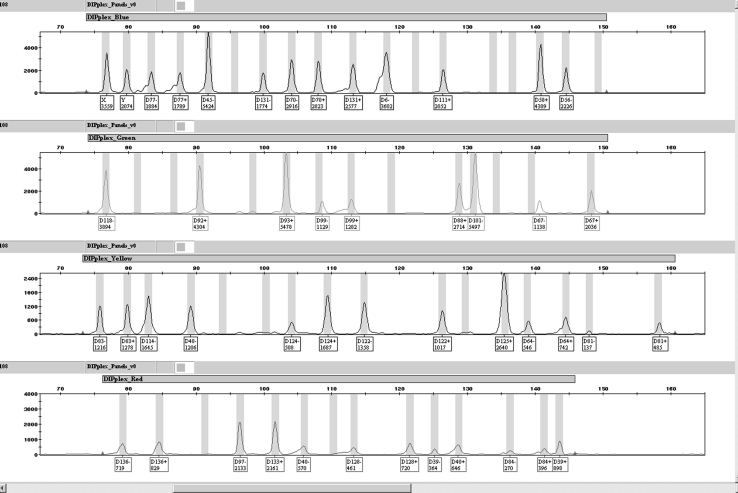
Representative DIPplex profile obtained from amplification of 100 pg DNA template

A representative DIPplex profile obtained from amplification of 100 pg DNA template is presented in Fig. 1. In the Polish population sample the INDELs frequency distributions showed no deviations from HWE (Bonferroni corrected, 0.0025 < *P* < 1.0000) evaluated by randomization procedure (10,000 cycles). Pairwise comparison using the exact test disequilibrium analysis with 16,000 permutation steps yielded departures from independence for 93 out of 435 pairs of INDELs under the analysis (0.0019 < *P* < 0.0480) (data not shown). The departures appeared statistically insignificant when the Bonferroni correction was used for the number of analysed loci. Observed heterozygosity for all the systems ranged 0.3525–0.5164, with an average of 0.4385, which is slightly lower than the values reported for Czech [6], German [13], Danish [14], Finnish [15], Central Spain, and the Basque Country populations [16]. In the Taiwanese population sample the INDELs frequency distributions showed no deviations from HWE (0.0032 < *P* < 1.0000) except for DLH39 (*P* = 0.0005). There were no statistically significant departures from independence between any pair-wise combination of INDELs (0.0018 < *P* < 0.0597) (data not shown). Observed heterozygosity for all the systems ranged 0.1270–0.6191, with an average of 0.4079, which corresponds to the values reported for Asian-Americans, and African-Americans [17]. The highest DP loci were HLD114 (DP = 0.660) for Poles and HLD118 (DP = 0.656) for Taiwanese. Based on data of the 30 INDELs the combined MP value among Poles amounts 7.98 × 10^−14^ which is more than two orders of magnitude lower than the value calculated for the Taiwanese population (1.22 × 10^−11^). Both parameters however, indicate a favourable value of a random match comparable with that of AmpFlSTR SGM kit [18, 19]. The combined values of PE are 0.9900 versus 0.9884, correspondingly (Table 1).

**Table 1:** Population data and forensic efficiency parameters for 30 DIPplex INDELs in Polish (*N*_P_ = 122) and Taiwanese (*N*_T_ = 126) population samples

	HLD	Chromosomal location	GenBank SNP ID	Poles								Taiwanese							
				-/DIP/	*P*	Ho	He	PIC	MP	PE	TPI	-/DIP/	*P*	Ho	He	PIC	MP	PE	TPI
1	77	7q31.1	rs1611048	0.4221	0.0859	0.4098	0.4899	0.37	0.352	0.115	0.84	0.5079	0.5971	0.4762	0.5019	0.37	0.364	0.168	0.95
2	45	2q31.1	rs2307959	0.4672	0.7044	0.4754	0.4999	0.37	0.366	0.167	0.95	0.3253	0.0417	0.5238	0.4408	0.34	0.449	0.209	1.05
3	131	7q36.2	rs1611001	0.3811	0.6875	0.4508	0.4767	0.36	0.382	0.148	0.91	0.7063	0.8310	0.4286	0.4165	0.33	0.432	0.132	0.88
4	70	6q16.1	rs2307652	0.5000	0.1113	0.4262	0.5021	0.38	0.346	0.131	0.87	0.3413	0.2355	0.3968	0.4514	0.35	0.390	0.112	0.83
5	6	16q13	rs1610905	0.4509	0.0025	0.3607	0.4972	0.37	0.340	0.096	0.79	0.4762	0.4729	0.5397	0.5009	0.37	0.398	0.225	1.09
6	111	17p11.2	rs1305047	0.4754	0.0181	0.3934	0.5008	0.37	0.340	0.110	0.82	0.8333	0.1096	0.2381	0.2789	0.24	0.569	0.041	0.66
7	58	5q14.1	rs1610937	0.5738	1.0000	0.4918	0.4911	0.37	0.382	0.180	0.98	0.5635	0.5866	0.5238	0.4939	0.37	0.396	0.209	1.05
8	56	4q25	rs2308292	0.3320	0.6944	0.4262	0.4481	0.35	0.401	0.125	0.86	0.3810	1.0000	0.4762	0.4735	0.36	0.392	0.168	0.95
9	118	20p11.1	rs16438	0.5902	0.0975	0.4098	0.4857	0.37	0.359	0.125	0.86	0.0953	0.0139	0.1270	0.1730	0.16	0.725	0.013	0.57
10	92	11q22.2	rs17174476	0.5820	0.1844	0.4262	0.4886	0.37	0.360	0.131	0.87	0.5317	0.1494	0.4286	0.5000	0.37	0.349	0.132	0.88
11	93	12q22	rs2307570	0.4631	1.0000	0.5000	0.4993	0.37	0.378	0.188	1.00	0.4206	0.4664	0.5238	0.4893	0.37	0.400	0.209	1.05
12	99	14q23.1	rs2308163	0.4057	1.0000	0.4836	0.4842	0.37	0.385	0.174	0.97	0.1587	0.0032	0.1905	0.2681	0.23	0.597	0.027	0.62
13	88	9q22.32	rs8190570	0.5697	0.3678	0.4508	0.4923	0.37	0.364	0.148	0.91	0.4524	0.0301	0.3968	0.4974	0.37	0.344	0.112	0.83
14	101	15q26.1	rs2307433	0.4180	0.5856	0.4590	0.4886	0.37	0.370	0.154	0.92	0.5397	0.2804	0.4444	0.4978	0.37	0.360	0.143	0.90
15	67	5q33.2	rs1305056	0.3934	0.0450	0.3852	0.4775	0.36	0.361	0.110	0.82	0.3413	0.6911	0.4286	0.4514	0.35	0.397	0.132	0.88
16	83	8p22	rs2308072	0.5287	0.8519	0.5164	0.5004	0.37	0.385	0.202	1.03	0.5794	0.1457	0.5556	0.4893	0.37	0.420	0.241	1.13
17	114	17p13.3	rs2307581	0.6885	0.3900	0.3934	0.4307	0.34	0.410	0.110	0.82	0.7143	1.0000	0.4127	0.4098	0.32	0.435	0.122	0.85
18	48	2q11.2	rs28369942	0.4385	0.1988	0.4344	0.4945	0.37	0.356	0.136	0.88	0.6667	1.0000	0.4444	0.4462	0.35	0.407	0.143	0.90
19	124	22q12.3	rs6481	0.3606	0.4328	0.4262	0.4631	0.35	0.385	0.131	0.87	0.4641	0.2831	0.5556	0.5015	0.37	0.408	0.241	1.13
20	122	21q22.11	rs8178524	0.5451	0.7106	0.5164	0.4980	0.37	0.388	0.202	1.03	0.8333	0.7486	0.2698	0.2789	0.24	0.562	0.052	0.68
21	125	22q11.23	rs16388	0.5779	0.8525	0.4754	0.4886	0.37	0.379	0.174	0.97	0.5238	0.5990	0.4762	0.5009	0.37	0.365	0.168	0.95
22	64	5q12.3	rs1610935	0.4631	0.1375	0.4262	0.4987	0.37	0.347	0.125	0.86	0.1429	1.0000	0.2540	0.2459	0.21	0.598	0.046	0.67
23	81	7q21.3	rs17879936	0.5574	0.4697	0.4590	0.4954	0.37	0.364	0.154	0.92	0.2778	0.0069	0.3016	0.4028	0.32	0.434	0.064	0.72
24	136	22q13.1	rs16363	0.5328	1.0000	0.4918	0.4999	0.37	0.373	0.180	0.98	0.5635	0.0062	0.6191	0.4939	0.37	0.464	0.314	1.31
25	133	3p22.1	rs2067235	0.4877	0.1463	0.4344	0.5018	0.37	0.349	0.136	0.88	0.6429	1.0000	0.4603	0.4610	0.35	0.398	0.155	0.93
26	97	13q12.3	rs17238892	0.5041	0.0728	0.4180	0.5020	0.37	0.364	0.148	0.91	0.6111	0.7100	0.4603	0.4772	0.36	0.382	0.155	0.93
27	40	1p32.3	rs2307956	0.4959	0.2106	0.4426	0.5021	0.37	0.353	0.145	0.90	0.3571	0.1250	0.3968	0.4610	0.35	0.388	0.116	0.84
28	128	1q31.3	rs2307924	0.5451	0.0175	0.3852	0.4980	0.37	0.341	0.107	0.82	0.6508	0.0775	0.3810	0.4563	0.35	0.380	0.106	0.82
29	39	1p22.1	rs17878444	0.5820	0.0059	0.3525	0.4842	0.37	0.347	0.083	0.76	0.8254	*0.0005*	0.1905	0.2894	0.25	0.576	0.027	0.62
30	84	8q24.12	rs3081400	0.4631	0.1550	0.4344	0.5012	0.37	0.364	0.148	0.91	0.2698	0.0390	0.3175	0.3956	0.32	0.440	0.071	0.73

*-/DIP/* frequency of deletion allele, *P* probability value for HWE, *H*_*o*_ observed heterozygosity, *H*_*e*_ expected heterozygosity, *PIC* polymorphic information content, *MP* match probability, *PE* power of exclusion, *TPI* typical paternity index

A pairwise testing for heterogeneity using the χ^2^-test was applied to compare allelic distributions. Minor or no significant differences were found between the Polish sample and Czech [6], Danish [14], Finnish [15], and American-Caucasian [17] data sets. Correspondingly, the comparison between the Taiwanese sample and Asian-Americans [17] yielded no significant differences (0.032 < *P* < 1.000). On the other hand, among differences revealed between the Poles and the Taiwanese at 14 INDELs (*P* < 0.05), these at HLD131, HLD111, HLD118, HLD99, HLD48, HLD122, HLD64, HLD81, HLD39, and HLD84 remained significant after the critical value was corrected for multiple testing (Table 2). It is noteworthy that the same loci significantly accounted for diversity between Caucasian and Asian samples, based on North American datasets published elsewhere [17].

**Table 2:** *P* values of population differentiation tested by an exact test and population specific *F*_ST_ indices per polymorphic locus (absolute values)

HLD	*P* value	*F*_ST_ Poles	*F*_ST_ Taiwanese	Average *F*_ST_
77	0.202	0.0108	0.0107	0.0107
45	0.043	0.0371	0.0376	0.0373
131	*0.000*	0.1893	0.1897	0.1895
70	0.022	0.0463	0.0468	0.0466
6	0.771	−0.0027	−0.0028	−0.0028
111	*0.000*	0.2450	0.2468	0.2459
58	0.887	0.0331	0.0331	0.0331
56	0.460	0.0013	0.0011	0.0012
118	*0.000*	0.4260	0.4282	0.4271
92	0.477	0.0011	0.0010	0.0011
93	0.569	−0.0004	−0.0003	−0.0004
99	*0.000*	0.1361	0.1381	0.1371
88	0.120	0.0232	0.0232	0.0232
101	0.089	0.0253	0.0252	0.0253
67	0.463	0.0017	0.0019	0.0018
83	0.477	0.0011	0.0012	0.0012
114	0.758	−0.0026	−0.0024	−0.0025
48	*0.001*	0.0962	0.0966	0.0964
124	0.151	0.0440	0.0437	0.0439
122	*0.000*	0.1732	0.1751	0.1741
125	0.394	0.0019	0.0018	0.0019
64	*0.000*	0.2132	0.2154	0.2143
81	*0.000*	0.1452	0.1459	0.1455
136	0.670	−0.0022	−0.0021	−0.0021
133	0.032	0.0438	0.0441	0.0440
97	0.118	0.0189	0.0191	0.0190
40	0.046	0.0346	0.0349	0.0347
128	0.113	0.0189	0.0192	0.0190
39	*0.000*	0.1287	0.1305	0.1296
84	*0.000*	0.0733	0.0742	0.0737

*Italicised* significant differentiation test *P* values, after Bonferroni correction

Wright’s *F*_ST_ was analysed to measure population substructure effects [20]. AMOVA results revealed that most of the molecular variation was due to variation within the analysed populations (92.54 %) rather than among them, with average fixation index values of 0.0743 and 0.0749 (Poles and Taiwanese, respectively). Our findings correspond to those presented by other authors who used AMOVA to compare the allelic frequencies for each DIPplex locus in populations of Europe, Africa, Asia and North America [16, 17]. Moreover, in our analysis individual INDELs displayed noticeable disparities in fixation index spanning from −0.0004 to −0.0003 (HLD93) to 0.4260 and 0.4282 (HLD118) for Poles and Taiwanese, respectively (Table 2). The individual mutation rate of a locus is one of the factors that may explain the observed discrepancy [21]. However, when compared with mutation rates of 10^−3^–10^−5^ for STRs [22, 23], SNPs have essentially mutation rates estimated at as low as 10^−8^ [24]. From the point of view of forensic genetics, markers with high heterozygosity and very low *F*_ST_ are potentially advantageous due to relatively high discrimination efficiency irrespective of population of origin [24, 25]. High heterozygosity enhances the polymorphism information at each SNP and low *F*_ST_ diminishes the chance of interpopulation effects. Some SNPs are reported to have remarkably little variation in allele frequency around the world [26]. On the other hand, ancestry informative single-nucleotide polymorphisms (AISNPs) are required to show low heterozygosity and high allele frequency divergence between different ancestral or geographically distant populations (*F*_ST_ values). These genetic markers are especially useful in establishing the high probability of an individual’s biogeographical ancestry [27, 28]. We have selected eight INDELs (HLD131, HLD111, HLD118, HLD99, HLD122, HLD64, HLD81, HLD39) with *F*_ST_ higher than 0.1 between Poles and Taiwanese as potential AISNPs for further analyses. Other sets of population data are needed to verify the robustness of these loci.
